# Delta-radiomics analysis based on magnetic resonance imaging to identify radiation proctitis in patients with cervical cancer after radiotherapy

**DOI:** 10.3389/fonc.2025.1523567

**Published:** 2025-01-29

**Authors:** Jing Xue, Menghan Wu, Jing Zhang, Jiayang Yang, Guannan Lv, Baojun Qu, Yanping Zhang, Xia Yan, Jianbo Song

**Affiliations:** ^1^ Third Hospital of Shanxi Medical University, Shanxi Bethune Hospital, Shanxi Academy of Medical Sciences, Tongji Shanxi Hospital, Taiyuan, China; ^2^ Research Centre for Nuclear and Radiation Frontier Technology, China Institute for Radiation Protection, Taiyuan, China; ^3^ Shanxi Bethune Hospital, Shanxi Academy of Medical Sciences, Third Hospital of Shanxi Medical University, Tongji Shanxi Hospital, Taiyuan, China; ^4^ Gynecological Tumor Treatment Center, The Second People’s Hospital of Datong, Cancer Hospital, Datong, Shanxi, China; ^5^ Shanxi Provincial Key Laboratory for Translational Nuclear Medicine and Precision Protection, Taiyuan, China

**Keywords:** cervical cancer, radiation proctitis, prediction model, radiomics, delta radiomics

## Abstract

**Objectives:**

To develop a magnetic resonance imaging (MRI)-based radiomics model for predicting the severity of radiation proctitis (RP) in cervical cancer patients’ post-radiotherapy.

**Methods:**

We retrospectively analyzed clinical data and MRI images from 126 cervical squamous cell carcinoma patients treated with concurrent chemoradiotherapy. Logistic regression (LR), Pearson correlation coefficient, and least absolute shrinkage and selection operator (LASSO) methods were utilized to select optimal imaging features, leading to a combined prediction model developed using a random forest (RF) algorithm. Model performance was assessed using the area under the curve (AUC), DeLong test, calibration curve, and decision curve analysis (DCA), with Shapley Additive exPlanations (SHAP) values for interpretation.

**Results:**

The samples were split into training (70%) and validation (30%) sets. The delta-radiomics model, comprising 10 delta features, showed strong predictive performance (AUC: 0.92 for training and 0.90 for validation sets). A comprehensive model combining delta-radiomics with clinical features outperformed this, achieving AUCs of 0.99 and 0.98. DeLong’s test confirmed the comprehensive model’s statistical superiority, and both calibration curves and DCA indicated good calibration and high net benefit. Key features associated with RP included D_1cc_, T1_wavelet-LLL_glcm_MCC, D_2cc_, and T2_original_firstorder_90 Percentile.

**Conclusions:**

The MRI-based delta radiomics model shows significant promise in predicting RP severity in cervical cancer patients following radiotherapy, with enhanced predictive performance when combined with clinical features.

## 1 Introduction

According to the 2022 Global Cancer Statistics Report, there were 661,000 new cases of cervical cancer globally (3.3% of all cancer cases), and 348,200 deaths (3.6%), making cervical cancer the fourth most common malignancy among women worldwide, highlighting its significant impact on global public health (1, 2). Radiotherapy is indicated for patients at all stages, particularly those with contraindications to surgery or those in advanced stages of the disease. While radiotherapy is effective in treating cervical cancer, its side effects, particularly radiation proctitis (RP), pose a significant threat to patients’ quality of life. It is estimated that more than 75% of patients receiving pelvic radiotherapy experience symptoms of RP (3). RP is typically diagnosed based on clinical manifestations and endoscopic findings. The current treatment outcomes are suboptimal, with symptoms that may recur and severely impact quality of life. Therefore, there is an urgent need for a non-invasive, accurate diagnostic tool to effectively assess and manage RP, thereby improving patients’ quality of life.

In recent years, radiomics has gained significant attention in the academic community as a novel research approach. Radiomics enables the extraction of numerous quantitative features from medical images, allowing for the mining of latent pathological information to achieve a more precise assessment of disease status. Le et al. developed a risk-scoring model based on 10 CT-based radiomics signatures for predicting overall survival in non-small cell lung cancer patients and achieved significant results (4). Building on this foundation, they further extended their study to explore the prediction of overall survival for different organs and cancer types, and similarly achieved excellent predictive performance (5). Similarly, in the area of radiation-induced adverse effects, Bao et al. successfully developed a predictive model for radiation encephalitis based on pre-treatment imaging features, further validating the potential of radiomics in clinical practice (6, 7). It is worth noting that the effect of tumor volume reduction before and after radiotherapy on adjacent organs at risk should not be overlooked. Consequently, recent studies have introduced a temporal dimension in the analysis of medical imaging data to better understand and predict disease progression. More precise and detailed delta-radiomics techniques have been explored in predicting radiation esophagitis, pneumonitis, and other conditions, demonstrating good predictive value (8–11). Currently, the use of delta-radiomics to predict RP in cervical cancer patients following radiotherapy remains a novel field.

Thus, in this study, we utilized delta-radiomics to comprehensively analyze MRI data before and after radiotherapy, in addition, through integrating these findings with clinical features, we constructed a new model for early prediction of RP, ultimately enabling early identification and individualized intervention measures.

## 2 Materials and methods

### 2.1 Basic information and grouping of patients

This study collected cervical cancer patients who received radiotherapy in Shanxi Bethune Hospital from May 2018 to October 2023. Inclusion criteria included: (a) patients with a newly diagnosed diagnosis of cervical squamous cell carcinoma (IB-IVA) without surgical treatment; (b) all patients were first-time receiving radiotherapy; (c) the patient has complete pathological, radiographic, and radiotherapy dose information; (d) patients who are clinically followed up and diagnosed with concurrent RP; (e) all patients were in good essential condition and had no apparent abnormalities in the rectum after MRI examination before radiotherapy. Some patients were excluded according to the following criteria: (a) a history of other malignancies; (b) lack of pathological, radiographic, and radiotherapy dose data of patients; (c) intolerance to radiotherapy or chemotherapy and failure to complete the treatment plan due to severe acute toxicity during treatment. Finally, 126 of the 356 patients met the criteria and were included in the study.

**Figure 1:**
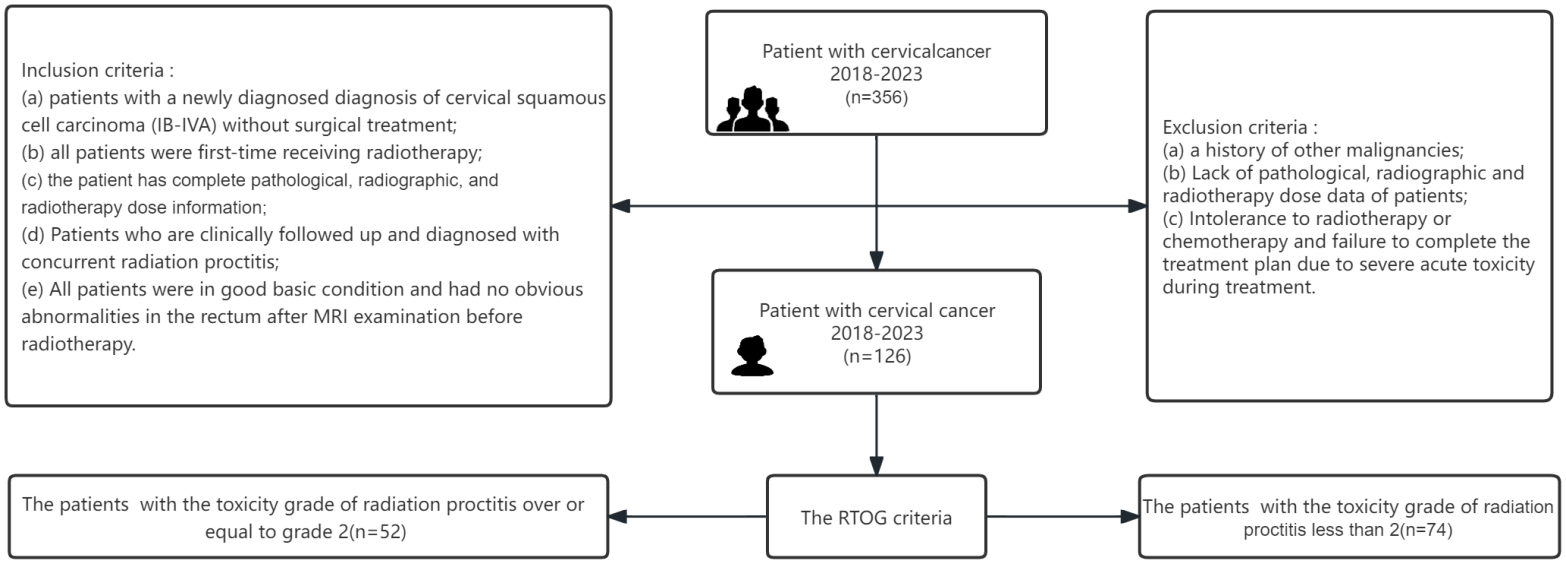
Flow diagram of the study enrolment patients. RTOG, Radiation Therapy Oncology Group.

We followed up the above patients for 1 year and graded the rectal injury of 126 patients according to the European Organization for Research and Treatment of Cancer-Radiation Therapy Oncology Group(RTOG/EORTC) classification criteria (12). We divided the patients into two groups: proctitis symptoms (grade 2-5), and no obvious proctitis symptoms (grade 0-1) (**Figure 1**).

### 2.2 Treatment options and data collection

All patients received external beam radiotherapy(EBRT)(1.8 ~ 2.0Gy/d, 5 times a week) and brachytherapy(BT)(5-7Gy/time, 4-6 times in total).MRI image data and clinical information of the above patients before radiotherapy and within 12 months after radiotherapy were collected, including age, the International Federation of Gynecology and Obstetrics (FIGO) staging of cervical cancer, minimum lymphocyte count value during treatment, radiation dosimetry parameters, the body mass index (BMI) and comorbidity. Among them, the radiation dosimetric parameters specifically include physical parameters related to external irradiation: the maximum dose (D_max_), the minimum dose (D_min_) received by the rectum, and the percentage of the total volume (V_30_, V_40_, V_45_) irradiated by 30Gy, 40Gy and 45Gy. In order to solve the problem of calculating the equivalent dose of different fractionated radiotherapy, the equivalent dose in 2Gy/f (EQD2) is finally used as the standard equivalent dose (refer to the following formula):


EQD2=nd(d+a/β)/(2+α/β)

Among them, n is the number of treatments, d is the fractional dose, 10 is the tumor in the a/β value, and 3 is the normal tissue (13). Finally, the minimum dose (D_1cc_, D_2cc_) accepted by the volume of 1cm^3^ and 2cm^3^ rectum after the superposition of internal and external irradiation is calculated.

### 2.3 Image acquisition and segmentation

All MRI images were obtained on a clinical whole-body 3.0T MRI scanner (GE Signa HDXT 3.0T MRI, GE Healthcare, USA) using a phased array 8-channel sensitivity coded abdominal coil. The patient took a supine position to maintain respiratory control. The scanning range covered the entire pelvis, from the upper edge of the iliac crest to the lower edge of the pubic symphysis. Sagittal T1 and T2 images were obtained from the picture archiving and communication system (PACS, Carestream, Canada) and exported in DICOM format. Tumor segmentation was performed manually using ITK-SNAP software, and the entire rectal region on the patient’s MRI image was defined as the region of interest (ROI). To reduce interfering information, a radiologist with 20 years of experience manually outlined the target region to include only structures within the rectum, excluding peripheral vasculature, peripheral tissues, and peripheral organs. Pelvic MRI images of 30 randomly selected patients were re-segmented by another radiologist with 20 years of experience. 2 weeks later, the first radiologist again re-outlined these randomly selected 30 images. Both radiologists were unaware of the patient’s clinical history and pathological information. The repeatability of segmentation was assessed using the intragroup correlation coefficient (ICC), and an ICC >0.8 was considered a good segmentation agreement (14).

### 2.4 Delta-radiomics features extraction and selection

Prior to feature extraction, the images were preprocessed to minimize variations due to different MRI scanners. All images were Z-value normalized to ensure that the images had a standard normal distribution, and then the images were resampled to a voxel size of 1 x 1 x 1 mm. Before the analysis, we first use the synthetic minority over-sampling technique (Smote) to preprocess the training set data so that the proportion of cases with different labels in the training set is 1:1. Then the variables with zero variance are excluded from the analysis, and the median is used to replace the missing values. Finally, the data is standardized. The pyradiomics in Python is used to extract imaging features, including first order features, shape features, texture features and transform features. After the feature extraction is completed, the patient label of ≥ 2 level is set to ‘ 1 ‘, and the patient label of < 2 level is set to ‘ 0 ‘. Then, the following formula is used to calculate the absolute change of features on the image after and before radiotherapy: delta-radiomics features represented by △RF. The calculation formula is as follows:


△RF=RFMRI2−RFMRI1

RF_MRI2_ represents the radiomics features of MRI images within 12 months after radiotherapy, and RF_MRI1_ represents the radiomics features of MRI images before radiotherapy.

**Figure 2:**
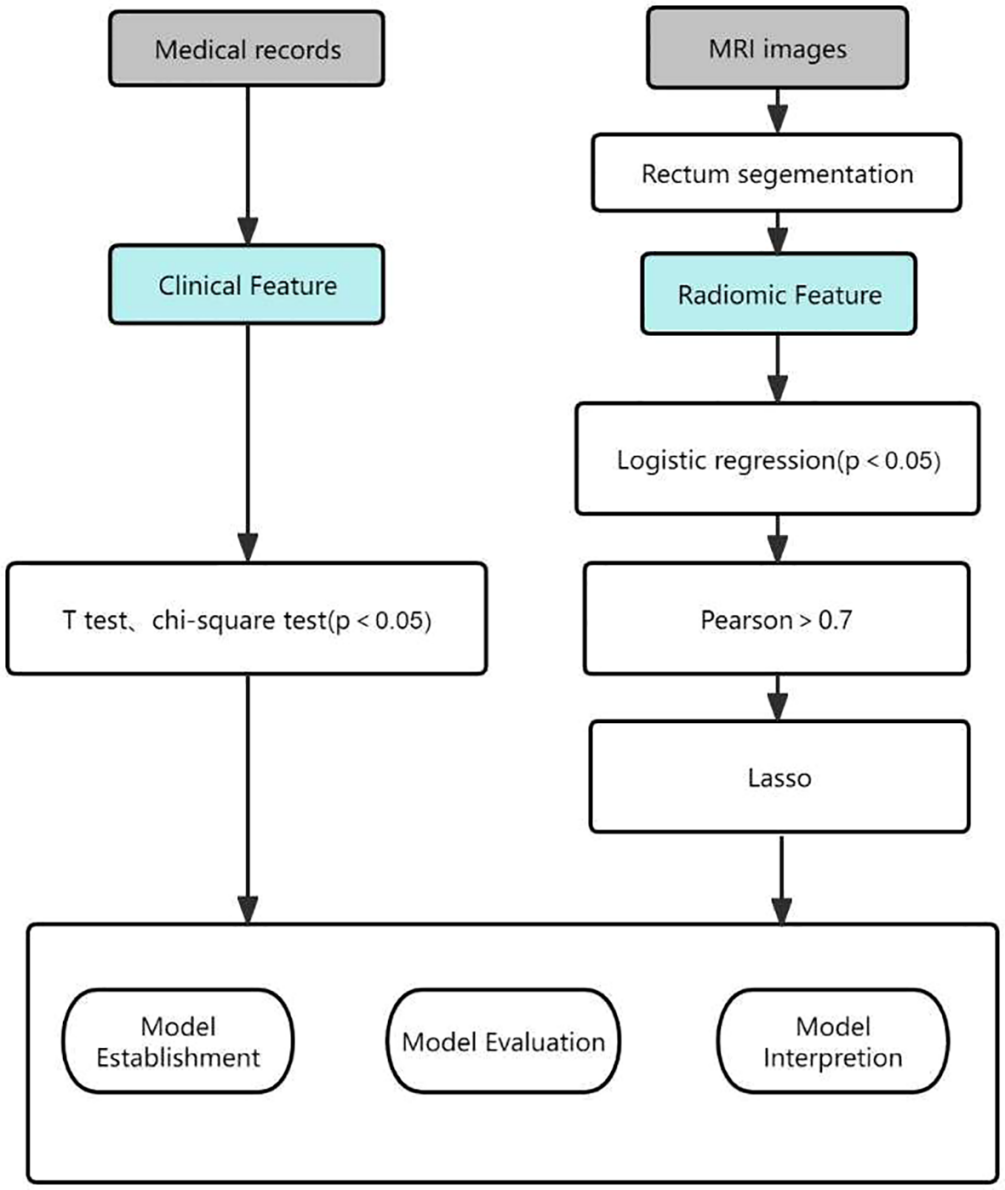
Workflow of the radiomic model development and model analysis process. LASSO, least absolute shrinkage and selection operator.

Statistical analyses of the extracted raw features were performed using Python, starting with feature dimensionality reduction using logistic regression (LR) analysis to select features that were significantly different between the two groups. The p-value is usually set at p < 0.2 but can also be set at p < 0.05 or p < 0.1. This requires the researcher to adjust the p-value according to the sample size (15). Due to the limited amount of data in this study, we set p < 0.05 as the threshold. This was then filtered using pairwise Pearson correlation analysis, which sets the Pearson correlation coefficient to 0.7, i.e., any two features were correlated. When their correlation coefficient is greater than 0.7, a feature is removed (16). Finally, to further reduce overfitting and selection bias, the least absolute shrinkage and selection operator (LASSO) was chosen to reduce the coefficients of most of the uncorrelated Δ RFs to zero once the optimal λ had been determined by 5-fold cross-validation, where the maximum area under the curve is the final value of λ. The coefficients of the Δ RFs were then reduced to zero (17).The process of generating and selecting radiomic features was illustrated in **Figure 2**.

### 2.5 Machine learning modeling

Random forest (RF) was used to construct the traditional radiomics model and delta-radiomics model. A clinical model was constructed based on the selected clinical features. Finally, statistically significant clinical indicators were included in the radiomics and delta-radiomics models, and the imaging + clinical joint model was constructed, respectively.

### 2.6 Statistical analysis

SPSS 26.0 software was used for statistical analysis. Quantitative data conforming to normal distribution were expressed as $\bar{\text{x}}$ ± s. T validation was used to compare the differences between groups. Qualitative data were expressed as frequency (%), and the χ2 validation was used to compare the differences between groups. Python 3.10 was used for feature screening and RF model establishment. The receiver operating characteristic (ROC) curve was used to judge the performance of the machine learning model, and the area under the curve (AUC), sensitivity and specificity were calculated. The DeLong validation is used to evaluate the performance differences of different models in the classification task, and the prediction model’s calibration curve and decision curve analysis (DCA) are constructed. Finally, the Shapley Additive Explanations (SHAP) values are used to analyze the prediction results of the model. P < 0.05 was considered statistically significant.

## 3 Results

### 3.1 Baseline information of patients

All samples were randomly divided into training and validation sets in a 7:3 ratio. After processing the training set data using the SMOTE method, 16 samples labeled as ‘1’ were added. The final training set included 104 cases (52 cases in the ‘1’ group and 52 cases in the ‘0’ group), while the validation set contained 32 cases (16 cases in each group). There were no significant differences in baseline characteristics (e.g., age, BMI, comorbidity, etc.) between the two groups (all p-values > 0.05), indicating that the groups were well-balanced at the start of the study (**Table 1**).

**Table 1:** Clinical features in the training and validation sets.

Variables		Training set (n=104)	Validation set (n=32)	p-Value
Age(years)		61.38 ± 10.12	59.37 ± 9.79	0.293
FIGO staging (%)	I	8(7.7%)	0(0.0%)	0.273
	II	48(46.2%)	19(59.4%)	
	III	41(39.4%)	12(37.5%)	
	IV	7(6.7%)	1(3.1%)	
Minimum lymphocyte count (x10^9^/L)		0.31 ± 0.15	0.33 ± 0.91	0.053
BMI (kg/m^2^)		20.90 ± 2.65	20.24 ± 2.38	0.899
Comorbidity (%)	Hypertension	54(51.9%)	15(46.9%)	0.617
	Diabetes	61(58.7%)	17(53.1%)	0.580
V_30_(%)		70.99 ± 13.76	69.91 ± 15.66	0.692
V_40_(%)		51.26 ± 12.79	55.90 ± 14.73	0.069
V_45_(%)		16.28 ± 16.34	20.46 ± 21.20	0.276
D_max_(Gy)		52.54 ± 4.46	52.54 ± 4.47	0.998
D_min_(Gy)		13.56 ± 8.97	12.07 ± 8.85	0.381
D_1CC_(Gy)		81.35 ± 3.66	80.80 ± 4.08	0.441
D_2CC_(Gy)		77.66 ± 3.59	77.16 ± 4.30	0.487

FIGO, the International Federation of Gynecology and Obstetrics; BMI, body mass index; V_30_, V_40_, V_50_, the percentage of the total volume irradiated by 30Gy, 40Gy and 45Gy; D_max_, the maximum dose; D_min_, the minimum dose; D_1cc_, D_2cc_, the dose accepted by the volume of 1cm^3^ and 2cm^3.^ p <0.05 was considered statistically significant.

To identify characteristics associated with the severity of RP, we conducted t-tests and χ² tests. The results indicated significant differences in dosimetric parameters, including V_40_, V_45_, D_max_, D_1cc_, D_2cc_, and the minimum lymphocyte count, across different severity groups (p < 0.05). Specifically, a lower minimum lymphocyte count correlated with increased severity of RP, while lower values of parameters such as V_40_ and D_max_ were associated with milder RP. Notably, V_30_, D_min_, age, FIGO stage, BMI, and comorbidity did not demonstrate statistically significant differences (**Table 2**).

**Table 2:** Comparison of the clinical features of different severities of RP.

Variables		<Grade 2 (n=68)	≥Grade 2 (n=68)	p-Value
Age(years)		60.92 ± 10.06	60.94 ± 9.70	0.990
FIGO staging	I	4(5.9%)	4(5.9%)	0.139
	II	28(41.2%)	39(57.4%)	
	III	33(48.5%)	20(29.4%)	
	IV	3(4.4%)	5(7.4%)	
Minimum lymphocyte count (x10^9^/L)		0.74 ± 0.30	0.52 ± 0.25	**0.047**
BMI (kg/m^2^)		21.09 ± 2.75	20.65 ± 2.53	0.366
Comorbidity (%)	Hypertension	36(25.9%)	33(48.5%)	0.383
	Diabetes	39(57.4%)	29(42.6%)	0.607
V_30_(%)		69.84 ± 15.15	71.31 ± 12.39	0.563
V_40_(%)		50.43 ± 16.47	55.87 ± 11.72	**0.028**
V_45_(%)		14.52 ± 17.99	22.40 ± 18.20	**0.018**
D_max_(Gy)		51.33 ± 4.81	54.02 ± 3.32	**<0.01**
D_min_(Gy)		11.81 ± 8.71	13.74 ± 8.95	0.231
D_1CC_(Gy)		80.31 ± 3.94	82.21 ± 2.92	**<0.01**
D_2CC_(Gy)		76.34 ± 3.90	78.93 ± 2.98	**<0.01**

RP, radiation proctitis; FIGO, the International Federation of Gynecology and Obstetrics; BMI, body mass index; V_30_, V_40_, V_50_, the percentage of the total volume irradiated by 30Gy, 40Gy and 45Gy; D_max_, the maximum dose; D_min_, the minimum dose; D_1cc_, D_2cc_, the dose accepted by the volume of 1cm^3^ and 2cm^3.^ p <0.05 was considered statistically significant.

Bolding represents p< 0.05, which is considered statistically significant.

### 3.2 Feature selection and machine establishment

A total of 1072 features were extracted from the T1 and T2 sequences before radiotherapy, with 12 features ultimately selected after a stepwise screening process. Similarly, 1072 features were extracted from the T1 and T2 sequences after radiotherapy, and 12 features were retained following gradual screening. Using the delta-radiomics calculation formula, a total of 1702 delta radiomic features (△RFs) were obtained, and 329 features remained after logistic regression analysis. Following Pearson correlation analysis, 64 features were retained, and finally, the top 10 radiomics features were selected using the LASSO.

**Figure 3:**
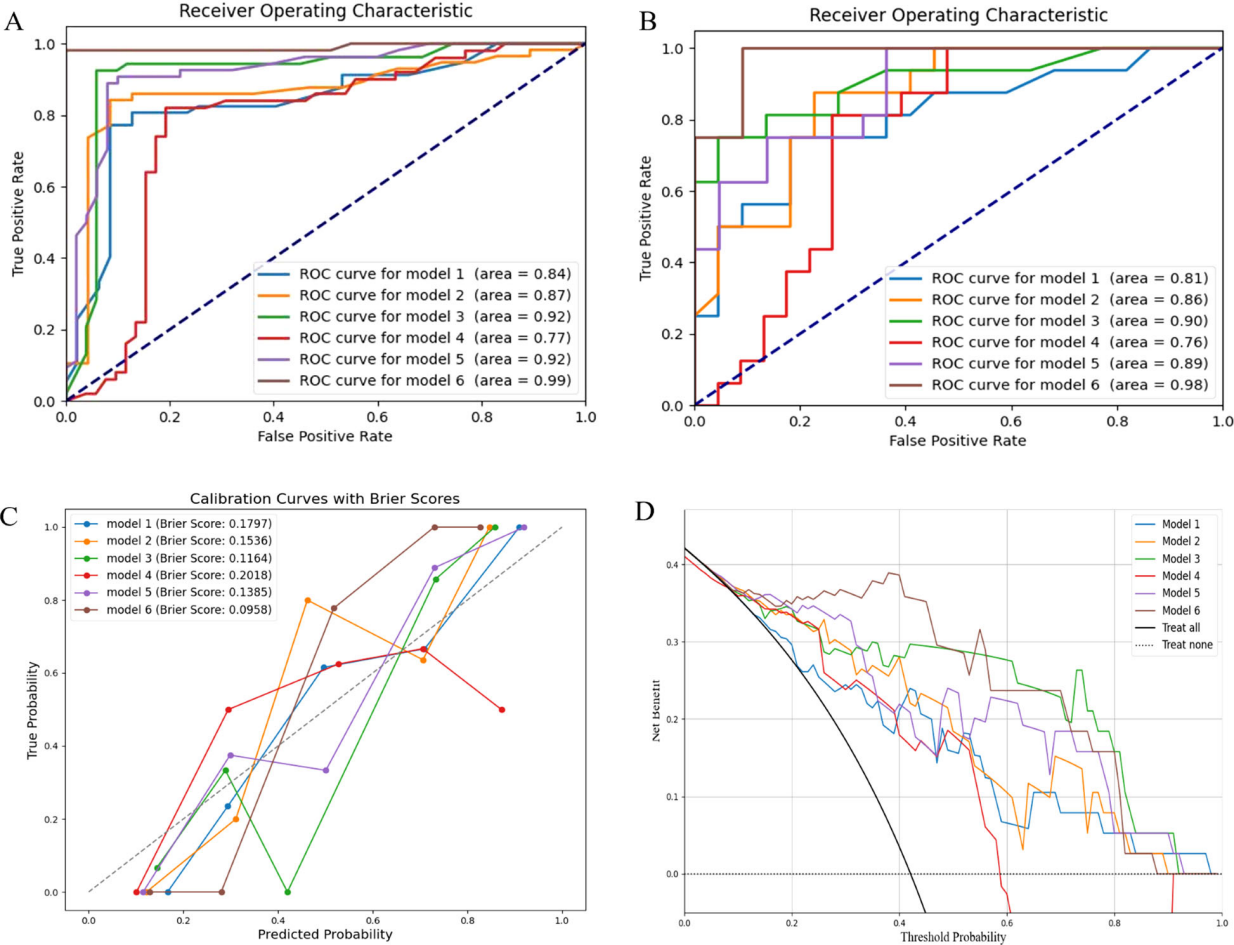
Model evaluation. **(A)** ROC curves of the training set; **(B)** ROC curves of the validation set; **(C)** Calibration curves of the radiomics models, the clinical model and the combined models; **(D)** DCA of the radiomics models, the clinical model and the combined models. ROC, the receiver operating characteristic; DCA, the decision curve analysis.

Subsequently, we used RF to develop six prediction models: the radiomics models (model 1, combining T1 and T2 sequences before radiotherapy; model 2, combining T1 and T2 sequences after radiotherapy; model 3, the delta model), the clinical model (model 4), the post-radiotherapy T1 and T2 sequences combined with clinical features (model 5), and the combined clinical and delta-radiomics models (model 6). In the training set, the predictive efficiency of the combined model, consisting of clinical indicators and radiomics features, outperformed the radiomics-only model, with an AUC, sensitivity, and specificity of 0.99, 0.981, and 1.000, respectively (**Figures 3A, B**). Similarly, in the validation set, the combined model outperformed the other models, and the AUC, sensitivity, and specificity data are provided in **Table 3**.

**Table 3:** Diagnostic efficacy of different RP prediction models in the training set and validation set.

	Model 1	Model 2	Model 3	Model 4	Model 5	Model 6
Training set						
AUC	0.840	0.870	0.920	0.770	0.920	0.990
Sensitivity	0.801	0.842	0.925	0.760	0.889	0.981
Specificity	0.872	0.915	0.941	0.808	0.920	1.000
Validation set						
AUC	0.810	0.860	0.900	0.760	0.890	0.980
Sensitivity	0.500	0.750	0.750	0.813	0.750	0.813
Specificity	0.909	0.818	0.955	0.739	0.864	0.909

RP, radiation proctitis; AUC, the area under the curve.

### 3.3 DeLong validation

We subsequently applied DeLong’s test to compare the differences in predictive power between the models. In the validation set, there were statistically significant differences in AUC between model 1, model 2, and model 6, whereas no significant differences were observed among the remaining models (**Table 4**).

**Table 4:** DeLong validation results between models.

Models	Training set		Validation set	
	Z	p-Value	Z	p-Value
Model 1 vs Model 2	-1.170	0.242(p>0.05)	-0.571	0.568(p>0.05)
Model 1 vs Model 3	1.048	0.295(p>0.05)	-1.099	0.272(p>0.05)
Model 1 vs Model 4	0.558	0.577(p>0.05)	-0.583	0.560(p>0.05)
Model 1 vs Model 5	-0.623	0.534(p>0.05)	-0.832	0.405(p>0.05)
Model 1 vs Model 6	0.147	0.883(p>0.05)	-2.312	**0.021(p<0.05)**
Model 2 vs Model 3	1.073	0.283(p>0.05)	-0.753	0.452(p>0.05)
Model 2 vs Model 4	0.232	0.816(p>0.05)	-0.018	0.986(p>0.05)
Model 2 vs Model 5	0.396	0.692(p>0.05)	-0.744	0.457(p>0.05)
Model 2 vs Model 6	-0.527	0.598(p>0.05)	-2.095	**0.036(p<0.05)**
Model 3 vs Model 4	-0.632	0.527(p>0.05)	0.610	0.542(p>0.05)
Model 3 vs Model 5	-1.605	0.101(p>0.05)	0.355	0.723(p>0.05)
Model 3 vs Model 6	-0.827	0.408(p>0.05)	-1.658	0.097(p>0.05)
Model 4 vs Model 5	0.398	0.691(p>0.05)	-0.291	0.771(p>0.05)
Model 4 vs Model 6	-0.094	0.691(p>0.05)	-1.833	0.067(p>0.05)
Model 5 vs model 6	0.907	0.364(p>0.05)	-1.896	0.058(p>0.05)

Model 1: the combining T1 and T2 sequences before radiotherapy; Model 2: the combining T1 and T2 sequences after radiotherapy; Model 3: the delta model; Model 4: the clinical model; Model 5: the post-radiotherapy T1 and T2 sequences combined with clinical features; Model 6: the combined clinical and delta-radiomics models. p <0.05 was considered statistically significant.

Bolding represents p< 0.05, which is considered statistically significant.

### 3.4 Model calibration curve and DCA

In the calibration curve, the Brier score is used to evaluate the accuracy of the prediction model. It was observed that the Brier score of model 6 was closest to 0, indicating the highest calibration (**Figure 3C**). DCA demonstrated that models 3 and 6 had significant clinical utility in predicting the severity of RP (**Figure 3D**).

### 3.5 Model interpretation results

**Figure 4:**
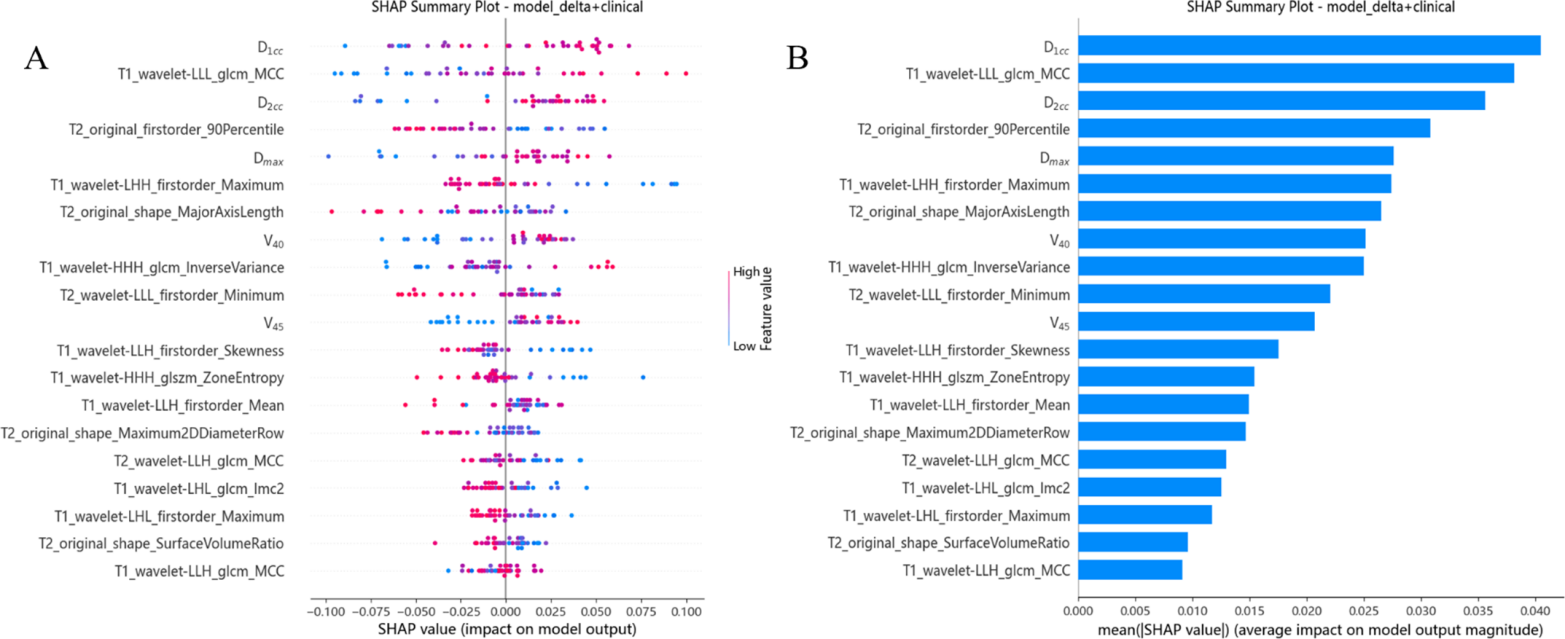
Model interpretation. **(A)** The beeswarm plot used SHAP values to show the distribution of each feature’s impacts. **(B)** The standard bar plot demonstrated the mean absolute value of the SHAP values for each feature.

We calculated and visualized SHAP values for each feature in the radiomics model. **Figure 4A** visually illustrates the 20 key features of the RF model, including 5 dosimetric parameters and 12 imaging features. Unique color dots indicate the impact of each feature: red indicates higher feature values, and blue indicates lower wind feature values. The bar chart in **Figure 4B** illustrates the average absolute value of the SHAP values for each feature. The above figure shows that D_1cc_ is the most essential feature for the RF model in predicting the severity of RP occurrence. The higher the feature values of D_1cc_, T1_wavelet-LLL_glcm_MCC, and D_2cc_, the greater the positive impact on the model output, while the lower the feature values T2_original_firstorder_90Percentile and T1_wavelet-LHH_firstorder_Maximum, the greater the positive impact on the model output.

## 4 Discussion

Early prediction of the severity of adverse reactions following radiotherapy for cervical cancer is crucial for adjusting treatment strategies. In this study, we developed and validated an MRI-based delta radiomics model to effectively predict the severity of RP. Furthermore, combining delta radiomics with clinical features enhanced the prediction performance compared to using delta radiomics alone. Therefore, a comprehensive prediction model integrating clinical and delta radiomics features provides an effective and non-invasive tool for guiding clinical decision-making.

In cervical cancer, radiomics has mainly been applied in staging, diagnosis, prognosis, adverse reactions, and lymph node metastasis (18, 19). For adverse reactions, researchers have used pre-radiotherapy computed tomography (CT) to predict the occurrence of RP, but the AUC of their radiomics model was only 0.71 (20). Subsequent studies shifted focus to MRI, where T2-weighted imaging (T2WI) sequences of pre-radiotherapy MRI were used to build a radiomics model with an AUC of 0.91, but the results were limited due to a small sample size and a lack of consideration for tumor volume regression (21). Our study aims to evaluate whether MRI features can effectively predict the occurrence of RP. Unlike previous studies, we focus on image information from the rectum, utilizing the commonly used T2WI sequence in combination with T1-weighted imaging (T1WI) to construct a multi-sequence radiomics model. Compared with a single sequence, this multi-sequence approach allows for a more comprehensive assessment of rectal edema, chronic inflammation, and fibrosis. Additionally, we applied delta-radiomics to dynamically monitor characteristic changes in longitudinal data at different time points. Although delta-radiomics is widely used for predicting cervical cancer prognosis, its use in predicting adverse reactions remains rare (22, 23). Wei et al. used LR to establish a model for predicting adverse reactions after radiotherapy for cervical cancer, achieving an AUC of 0.6855 (15). With advancements in machine learning, Xie et al. employed support vector machines (SVM), LR, and RF to establish a model for RP prediction, with RF showing superior performance over other models (AUC: 1.000, 0.713, 0.820, 0.798) (24, 25). Based on these studies, we selected the delta-radiomics model developed using RF, which achieved good predictive performance (AUC: 0.92, 0.90) and holds promise as a potential imaging biomarker for RP.

In addition to imaging features, previous studies have shown that risk factors for RP following radiotherapy for cervical cancer include patient age, clinical stage, radiotherapy method, and dose (26–28). Fang et al. further indicated that vascular conditions, such as hypertension, diabetes, and atherosclerosis, are also potential risk factors (29, 30). In our study, we found that traditional clinical characteristics, such as age, BMI, and comorbidity, were not statistically significant for RP (p > 0.05), which may be related to individual variability and post-treatment care (31, 32). In order to further enrich the clinical model, we obtained parameters from the DVH map, especially combining the parameters D_1cc_ and D_2cc_ of internal and external irradiation doses were correlated with the severity of RP (p < 0.05), which was consistent with the findings of Y (33–36). For patients receiving conventional radiotherapy, treatment is often interrupted due to myelosuppression, with lower radiation doses causing transient suppression and higher doses potentially leading to irreversible damage, emphasizing the influence of immunity on prognosis (37–39). At present, most studies focused on the effect of changes in the immune system on the prognosis of cervical cancer during radiotherapy (40, 41). Our study shifted focused to the relationship between the immune system and the toxic response to cervical cancer after radiotherapy and found that the minimal lymphocyte count was significantly correlated with RP (p < 0.05), making it a significant predictor. It has gradually become one of the important predictors in head, neck and pelvic malignant tumors (42, 43). Other studies also incorporated factors such as vitamin D, radiotherapy interval, and microbial characteristics to enrich the predictive results (44–46), and the influencing factors of RP can be further explored based on this in the future.

Neither imaging features nor clinical indicators alone can fully capture the specific effects of radiotherapy on rectal tissue. Therefore, in order to more comprehensively understand the changes of patients during radiotherapy and formulate more accurate intervention measures, we established a clinically significant feature model combined with imaging features. The ROC curve demonstrated that the efficiency of the simple radiomics model was inferior to that of the combined model. Subsequently, DeLong’s test confirmed that the delta-combined clinical radiomics model was statistically superior to the radiomics model established at a single time point. Calibration curve and DCA also indicated that the combined model had superior calibration and net benefit.

Although our models show some advantages, one of the key factors in determining whether a physician will adopt a machine learning model to aid in clinical decision-making is their ability to understand how the model arrived at its conclusions. In order to improve the transparency and credibility of the models, the researchers proposed interpretable machine learning models, such as for predicting mortality from gastrointestinal bleeding, the risk of new-onset atrial fibrillation in critically ill patients, and long-term clinical outcomes in patients with recurrent pericarditis (47–49). Based on the above studies, we plotted standard histograms in this study and found that texture features extracted by wavelet transform and grey level covariance matrix (GLCM) contributed the most to the joint model in imaging features. These texture features are effective in capturing the microscopic changes in tissues after radiotherapy, especially the imaging alterations caused by radiation damage. Zhou et al. showed that wavelet transform extracted texture features outperformed other features in predicting the response to neoadjuvant chemotherapy in patients with advanced breast cancer and that these features have the potential to serve as potential biomarkers for predicting the response to chemotherapy (50). In our study, by quantifying these textural features, we found that their changes were closely associated with pathological changes such as edema, fibrosis and vascular proliferation of rectal tissue, thus providing a strong basis for predicting the severity of RP.

There are certain limitations to this study that need to be addressed. First, the sample size was small and from only a single research center, which may have led to a selection bias. Therefore, future studies should consider increasing the sample size, adopting a multi-center design, and conducting external validation to improve the generalizability and reliability of the results. Second, this study used a manual method of outlining ROI. Although this method can provide more accurate results, the process is time-consuming and challenging to apply efficiently in large-scale samples. Automated image segmentation techniques should be explored in the future to improve analysis efficiency and scalability. Finally, although this study used traditional machine learning methods for model development, there is still room for algorithm optimization and accuracy improvement. More advanced algorithms, such as deep learning, can be considered in the future to enhance the predictive performance of the model and avoid the overfitting problem, especially with the support of multi-center data, which further optimize the RP prediction model. Currently, our institute has initiated a related study to validate and optimize these improvements.

## 5 Conclusion

We developed and validated an MRI-based delta radiomics model to effectively predict the severity of RP. Furthermore, delta radiomics combined with clinical features improved predictive performance compared to delta radiomics alone. This confirms the important role of radiomics in predicting toxic reactions and provides scientific basis for early prediction and intervention in clinical practice.

## Data Availability

The original contributions presented in the study are included in the article/supplementary material. Further inquiries can be directed to the corresponding authors.
